# Body mass index and overall survival in patients with gastric cancer: stage-specific associations and prognostic implications

**DOI:** 10.3389/fnut.2026.1937418

**Published:** 2026-10-21

**Authors:** Sijing Zhou, Dong Yu, Xiaojie Wang, Zhouwei Zhan, Zengqing Guo, Jiami Yu

**Affiliations:** 1 Department of Medical Oncology, Clinical Oncology School of Fujian Medical University, Fujian Cancer Hospital, Fuzhou, China; 2 Department of Clinical Nutrition, Clinical Oncology School of Fujian Medical University, Fujian Cancer Hospital, Fuzhou, China; 3 Department of Radiology, Clinical Oncology School of Fujian Medical University, Fujian Cancer Hospital, Fuzhou, China; 4 Department of Medical Oncology, Clinical Oncology School of Fujian Medical University, Fujian Cancer Hospital, Fuzhou, China

**Keywords:** body mass index, gastric cancer, nomogram, nutritional status, prognostic prediction

## Abstract

**Introduction:**

Nutritional impairment is common in gastric cancer and may affect treatment outcomes and overall survival (OS). However, the prognostic significance of body mass index (BMI), particularly across disease stages, remains uncertain. This study aimed to evaluate the association between baseline BMI and OS, examine potential stage-specific differences, and assess the additional prognostic value of nutritional and functional indicators.

**Methods:**

This observational cohort study included 380 adults with histologically confirmed gastric cancer, categorized as underweight (BMI <18.5 kg/m^2^), normal weight (18.5–23.9 kg/m^2^), overweight (24.0–27.9 kg/m^2^), or obese (≥28.0 kg/m^2^). Associations with OS were evaluated using Kaplan–Meier analysis, multivariable Cox regression, BMI-by-stage interaction testing, parsimonious stage-specific sensitivity analyses, and restricted cubic splines. Prognostic performance was assessed using bootstrap-corrected concordance indices (C-indices) and time-dependent areas under the curve (AUCs).

**Results:**

Among 374 patients included in the primary multivariable analysis, 202 deaths occurred. After adjustment for clinical, nutritional, and treatment-related factors, overweight was associated with lower mortality than normal weight (hazard ratio [HR], 0.62; 95% confidence interval [CI], 0.41–0.93; *p* = 0.020), whereas underweight and obesity were not significantly associated with OS. The BMI-by-stage interaction was not significant (*p* = 0.173). In stage-specific sensitivity analyses, overweight was associated with lower mortality in stage III–IV disease (HR, 0.61; 95% CI, 0.41–0.93), whereas the early-stage estimate for underweight was imprecise (HR, 5.19; 95% CI, 1.42–18.96; 26 deaths). The extended clinical-nutritional model achieved an optimism-corrected C-index of 0.681 after 1,000 bootstrap resamples, compared with 0.646 for the clinical reference model. Its 1-, 3-, and 5-year time-dependent AUCs were 0.744, 0.748, and 0.702, respectively.

**Discussion:**

Overweight BMI was associated with lower mortality in patients with gastric cancer, while incorporating nutritional and functional indicators modestly improved prognostic discrimination. However, the nonsignificant BMI-by-stage interaction and limited early-stage events preclude definitive conclusions regarding effect modification by disease stage. These findings highlight the potential value of integrating BMI and nutritional assessments into prognostic evaluation.

## 1 Introduction

Gastric cancer remains a major global health burden despite improvements in screening, surgical techniques, systemic therapy, and supportive care. According to the Global Cancer Statistics (GLOBOCAN) 2022 estimates, nearly one million new cases of gastric cancer occurred worldwide, making it the fifth most commonly diagnosed malignancy, while gastric cancer remained among the leading causes of cancer-related death (1). Prognosis varies markedly according to tumor stage, treatment eligibility, and patient-related factors. Although tumor characteristics remain the dominant determinants of survival, increasing attention has been directed toward potentially modifiable host factors, particularly nutritional status, functional reserve, and systemic inflammation.

Nutritional impairment is particularly common in gastric cancer because both the disease and its treatment can interfere with food intake, digestion, and nutrient absorption. Patients may experience anorexia, early satiety, nausea, vomiting, obstruction, treatment-related toxicity, and progressive cancer-associated catabolism. These changes can result in weight loss, loss of skeletal muscle and adipose tissue, impaired physical function, reduced treatment tolerance, and worse clinical outcomes (2, 3). A recent systematic review reported a high prevalence of Patient-Generated Subjective Global Assessment (PG-SGA) -defined malnutrition among patients with gastrointestinal cancers, although estimates varied considerably across study populations and assessment settings (4). Malnutrition and weight loss have also been associated with poorer survival among patients with gastric cancer (5). Nutritional evaluation has therefore become an important component of comprehensive gastric cancer care. Several tools are available for this purpose, including Nutritional Risk Screening 2002 (NRS2002), the PG-SGA, blood biochemical indicators, and anthropometric measurements. However, these measures assess partly different dimensions of nutritional vulnerability and may not have equivalent value for long-term survival prediction.

Body mass index (BMI) is one of the most accessible anthropometric indicators in routine clinical practice. Low BMI may reflect inadequate nutritional intake, cancer-associated weight loss, cachexia, or limited physiological reserve. Conversely, moderately elevated BMI may indicate greater energy reserves and a higher capacity to tolerate surgery, systemic treatment, and progressive catabolic stress. Recent evidence suggests that underweight and weight loss are generally associated with poorer survival in patients with cancer, particularly those with gastrointestinal malignancies (6). In gastric cancer, several observational studies have similarly reported unfavorable outcomes among patients with low BMI or an apparent survival advantage among patients with relatively high BMI. For example, a large cohort of patients with stage II - III gastric cancer undergoing curative gastrectomy found that low BMI was associated with incomplete perioperative chemotherapy and poorer OS (7). Another postoperative study reported an apparent obesity paradox in gastric cancer, although the association was influenced by postoperative complications and clinical subgroup characteristics (8).

Nevertheless, the relationship between BMI and gastric cancer prognosis remains uncertain. BMI does not distinguish skeletal muscle from adipose tissue and cannot identify sarcopenia, sarcopenic obesity, visceral adiposity, edema, or recent involuntary weight loss. It must also be recognized that an apparently favorable association between elevated BMI and survival may also result from reverse causation, because patients with aggressive disease may lose weight before diagnosis and move from a previously higher BMI category into the normal-weight or underweight range. In upper gastrointestinal cancers, pre-diagnostic weight loss has been shown to provide prognostic information beyond BMI measured at the time of diagnosis (9). In addition, the association between BMI and survival may vary by tumor stage. Nutritional and metabolic reserves may be particularly relevant in advanced disease, in which systemic inflammation, metastatic burden, reduced intake, and tissue wasting are more prominent. However, stage-specific patterns of association have not been fully characterized.

It also remains unclear how the prognostic information provided by BMI relates to and interacts with that obtained from established nutritional assessment tools. NRS2002 is primarily designed to identify hospitalized patients at nutritional risk and guide the need for nutritional intervention, whereas PG-SGA provides a more comprehensive cancer-oriented assessment incorporating weight change, dietary intake, nutrition-impact symptoms, functional status, metabolic stress, and physical findings. Recent comparative evidence suggests that PG-SGA may identify nutritional impairment more comprehensively than NRS2002 in patients with cancer (10). However, nutritional screening, comprehensive nutritional assessment, and long-term prognostic prediction are distinct clinical objectives. Recent evidence indicates that PG-SGA-defined malnutrition is associated with adverse outcomes in patients with cancer (11), and both baseline PG-SGA status and longitudinal deterioration in PG-SGA scores may convey prognostic information. Whether BMI, NRS2002, and PG-SGA demonstrate similar or divergent associations with OS in gastric cancer therefore warrants further investigation.

In this study, we primarily aim to evaluate the association between baseline BMI and OS in patients with gastric cancer. The secondary aims were to examine BMI using predefined clinical categories and as a continuous exposure, evaluate stage-specific associations between BMI and mortality, compare survival patterns according to BMI, NRS2002, and PG-SGA, assess the prognostic relevance of functional, laboratory, tumor-related, and treatment-related characteristics, and explore the feasibility of combining nutritional and functional indicators in an individualized survival prediction model.

## 2 Methods

### 2.1 Study design and participants

This observational cohort study included adult patients newly diagnosed with gastric cancer at the Fujian Cancer Hospital between September 1, 1995, and May 25, 2017. Baseline was defined as the date of first histopathological diagnosis. Demographic, clinical, tumor-related, nutritional, treatment-related, and laboratory data were collected at the initial assessment. The primary objective was to evaluate the association between baseline BMI and OS; secondary analyses examined nutritional, inflammatory, functional, and stage-related patterns.

Patients were eligible if they met all of the following criteria: (1) age ≥18 years; (2) male or female sex; (3) newly diagnosed gastric cancer confirmed by histopathological examination; (4) availability of baseline height and weight measurements required for BMI calculation; (5) availability of baseline clinical and nutritional assessment data required for the principal analyses; and (6) provision of written informed consent for participation in the study.

Patients were excluded if they met any of the following criteria: (1) uncontrolled or severe cardiac, hepatic, pulmonary, or renal disease that could substantially affect nutritional status, treatment tolerance, or short-term survival independently of gastric cancer; (2) inability to complete the required baseline clinical or nutritional assessment; (3) absence of essential survival information, including survival time or vital status; or (4) refusal to participate or refusal to provide written informed consent.

For patients with incomplete information for individual variables, the number of observations available for the corresponding analysis was reported.

### 2.2 Baseline data collection

Baseline information was obtained from clinical assessments, medical records, nutritional evaluations, and laboratory examinations performed at or immediately following the first pathological diagnosis. Demographic and lifestyle variables included age, sex, family history of gastric cancer, smoking status, alcohol consumption, residential area, and educational level.

Tumor-related variables included baseline TNM clinical stage and histological differentiation. TNM stage was categorized as stages I, II, III, and IV. For prespecified stage-related analyses, stages I and II were combined as early-stage disease and stages III and IV as advanced-stage disease. Histological differentiation was based on the first-diagnosis grade; mixed grades were assigned to the highest recorded grade, and unknown grades were treated as missing data. The number of metastatic organs was excluded from all prognostic models because review of the source data showed that its timing could not be aligned reliably with the baseline assessment.

Treatment-related variables included surgical procedure, chemotherapy, immunotherapy, and nutritional support. Surgical management was categorized as radical surgery, palliative resection, or non-surgical treatment. Nutritional support was classified as no nutritional support, parenteral nutrition, enteral nutrition and/or oral nutritional supplements, or combined nutritional support. Chemotherapy was coded from the general chemotherapy variable and indicates treatment that had already been initiated by the baseline assessment; it does not specifically identify postoperative adjuvant treatment.

Functional status was evaluated using the Karnofsky Performance Status (KPS). Laboratory indicators included serum albumin (g/L), prealbumin (mg/L), hemoglobin (g/L), C-reactive protein (mg/L), platelet count (×10^9^/L), and the neutrophil-to-lymphocyte ratio (NLR). Available information on recent weight loss was also collected.

### 2.3 Assessment of body mass index

BMI was calculated as body weight in kilograms divided by the square of height in meters:


$$\mathrm{BMI}=\frac{\;\text{body weight}\;(\mathrm{Kg})}{\text{height}\;{\left(m\right)}^{2}}$$

For the primary categorical analysis, patients were classified into four groups directly from their recorded numerical baseline BMI:

- Underweight: BMI < 18.5 kg/m^2^;
- Normal weight: BMI 18.5–23.9 kg/m^2^;
- Overweight: BMI 24.0–27.9 kg/m^2^;
- Obesity: BMI ≥ 28.0 kg/m^2^.

Patients with a BMI of 18.5–23.9 kg/m^2^ were used as the reference group in the Cox regression analyses. BMI was additionally analyzed as a continuous variable using restricted cubic spline modeling to characterize its association with mortality risk without assuming a strictly linear relationship.

### 2.4 Nutritional assessment

Nutritional risk was assessed using the Nutritional Risk Screening 2002 (NRS2002). Patients with an NRS2002 score <3 were classified as having a low nutritional risk, whereas those with a score ≥3 were classified as being at nutritional risk.

Nutritional status was evaluated using the Patient-Generated Subjective Global Assessment (PG-SGA). The Chinese version of the PG-SGA used in this study has demonstrated acceptable reliability and validity in Chinese patients with gastric cancer (9). Patients were categorized into three groups according to their PG-SGA scores:

- 0–3 points: relatively stable nutritional status;
- 4–8 points: nutritional intervention indicated;
- ≥9 points: urgent nutritional intervention indicated.

The prognostic associations of NRS2002 and PG-SGA with OS were evaluated separately. Kaplan - Meier analyses were used to compare survival across the predefined categories, and the corresponding associations were further examined using Cox proportional hazards regression.

### 2.5 Weight-loss assessment

Available recalled weights during the preceding 1 and 6 months were collected. Weight change (%) = 100 × (current recalled weight − previous recalled weight) / previous recalled weight; negative values indicate weight loss. Previous weights recorded as zero were treated as missing. Significant weight loss was defined as a reduction of at least 5% over 6 months.

Because 6-month weight-loss information was available for only a subset of the study population, analyses involving this variable were considered exploratory.

### 2.6 Outcome definition

The primary outcome was OS, defined as the interval from the date of first histopathological diagnosis to death from any cause. Patients who remained alive were censored at their last documented contact. Follow-up ranged from 0.2 to 67.8 months; the reverse Kaplan–Meier estimate of median follow-up was 37.0 months.

### 2.7 Statistical analysis

Baseline characteristics were summarized according to BMI category. Continuous variables were presented as mean with standard deviation or median with interquartile range, as appropriate, and categorical variables as frequencies and percentages. Percentages use non-missing observations for each variable within each BMI group and are rounded independently to one decimal place; totals may therefore differ slightly from 100.0%. Available n is shown for incomplete variables. NA (*n* = 0) indicates that no observations were available, rather than a measured zero. *p* values are from Welch one-way ANOVA for mean ± SD rows, Kruskal-Wallis tests for median (IQR) rows, and Fisher–Freeman–Halton tests with 100,000 Monte Carlo samples (seed 20,260,911) for categorical variables. Comparisons of CRP and 6-month weight change include the three BMI groups with available observations.

Kaplan - Meier methods were used to estimate survival functions according to BMI, NRS2002, and PG-SGA categories. Differences between survival curves were evaluated using the log-rank test. Numbers at risk were reported beneath the Kaplan - Meier curves.

Univariable Cox proportional hazards regression was used to examine the association of individual demographic, tumor-related, nutritional, functional, laboratory, and treatment-related variables with OS. Effect estimates were reported as hazard ratios (HRs) with 95% confidence intervals (CIs). Analyses excluded missing values separately for each model. The surgery comparison was restricted to patients who underwent radical or palliative surgery. Tied event times were handled using the Efron method, and model-based Wald confidence intervals and *p* values were reported.

Variables identified from the univariable analyses and variables considered clinically relevant were evaluated using multivariable Cox proportional hazards regression. The final model included BMI category, age, TNM clinical stage, PG-SGA category, KPS, prealbumin, NLR, and baseline chemotherapy. Age was modeled flexibly with a restricted cubic spline using four degrees of freedom. Surgical procedure and nutritional support type were treated as stratification variables. The BMI category of 18.5–23.9 kg/m^2^, stage I disease, PG-SGA score of 0–3, and no chemotherapy were the reference categories. The final model used 374 complete cases, excluding six patients with missing TNM stage. The proportional hazards assumption was assessed for individual covariates and globally using score tests with the Kaplan–Meier transformation of time.

Effect modification by disease severity was evaluated formally by adding BMI-category-by-stage-group (I-II vs. III-IV) interaction terms to the final Cox model and comparing nested models with a likelihood-ratio test. A continuous BMI-by-stage interaction was examined as a sensitivity analysis. Because the early-stage subgroup contained only 26 deaths, stage-specific estimates were additionally evaluated in parsimonious sensitivity models that included BMI category and within-stratum TNM stage, with baseline hazards stratified by age group (<60, 60–69, or ≥70 years). These subgroup analyses were considered exploratory.

Restricted cubic spline models with four degrees of freedom characterized the continuous associations of BMI and serum prealbumin with mortality. A BMI of 22 kg/m^2^ and a prealbumin concentration of 194.5 mg/L were used as reference values. Overall association and nonlinearity were tested with likelihood-ratio tests, and adjusted HRs with 95% CIs were plotted across the observed ranges.

All statistical tests were two-sided, and a *p* value <0.05 was considered statistically significant.

### 2.8 Exploratory prognostic model

An exploratory extended clinical-nutritional model was constructed to estimate 1-, 3-, and 5-year OS. A conventional clinical reference model included age, sex, TNM stage, and baseline chemotherapy. The extended model added continuous BMI, PG-SGA score, KPS, serum prealbumin, and NLR. Age was modeled with a four-degree-of-freedom restricted cubic spline in both models. A nomogram was generated from the extended model.

Incremental model fit was assessed by a likelihood-ratio test comparing the nested clinical and extended models. Discrimination was quantified by Harrell’s C-index and time-dependent AUC at 1, 3, and 5 years. Internal validation used 1,000 bootstrap resamples to estimate optimism-corrected C-indices. Calibration was assessed by comparing mean predicted survival with Kaplan–Meier estimates within quintiles of predicted risk. Because this was an exploratory model without external validation, no claim of clinical utility was made.

## 3 Results

### 3.1 Patient characteristics

A total of 380 patients with gastric cancer were included. The mean age was 59.5 ± 11.6 years (range, 19–87 years). According to baseline BMI, 65 patients (17.1%) were underweight, 220 (57.9%) were of normal weight, 83 (21.8%) were overweight, and 12 (3.2%) were obese. The median BMI values were 17.1, 20.9, 25.2, and 29.3 kg/m^2^ in the respective groups (Table 1).

**Table 1:** Baseline characteristics.

Characteristic	<18.5 *N* = 65	18.5–23.9 *N* = 220	24.0–27.9 *N* = 83	≥28.0 *N* = 12	*p* value
BMI (kg/m^2^), median (IQR)	17.1 (16.3, 18.0)	20.9 (19.8, 22.4)	25.2 (24.4, 26.1)	29.3 (28.7, 30.2)	—
Age (years), mean ± SD	61.0 ± 10.5	59.3 ± 11.6	58.4 ± 12.0	61.4 ± 14.3	0.510
Sex, *n* (%)					0.932
Male	44 (67.7%)	155 (70.5%)	60 (72.3%)	9 (75.0%)	
Female	21 (32.3%)	65 (29.5%)	23 (27.7%)	3 (25.0%)	
TNM clinical stage, *n* (%) (*n* = 374)					0.164
Stage I	1 (1.6%)	18 (8.3%)	9 (11.0%)	1 (8.3%)	
Stage II	5 (7.8%)	36 (16.7%)	16 (19.5%)	3 (25.0%)	
Stage III	33 (51.6%)	89 (41.2%)	29 (35.4%)	5 (41.7%)	
Stage IV	25 (39.1%)	73 (33.8%)	28 (34.1%)	3 (25.0%)	
Available n	64	216	82	12	
Histological differentiation, *n* (%) (*n* = 236)					0.858
Well differentiated (G1)	0 (0.0%)	4 (2.7%)	0 (0.0%)	0 (0.0%)	
Moderately differentiated (G2)	11 (26.8%)	43 (28.9%)	16 (38.1%)	1 (25.0%)	
Poorly differentiated (G3)	30 (73.2%)	101 (67.8%)	26 (61.9%)	3 (75.0%)	
Undifferentiated (G4)	0 (0.0%)	1 (0.7%)	0 (0.0%)	0 (0.0%)	
Available n	41	149	42	4	
Family history of gastric cancer, yes, *n* (%)	11 (16.9%)	17 (7.7%)	10 (12.0%)	1 (8.3%)	0.147
Smoking, yes, *n* (%)	35 (53.8%)	87 (39.5%)	30 (36.1%)	5 (41.7%)	0.147
Alcohol consumption, yes, *n* (%)	10 (15.4%)	33 (15.0%)	15 (18.1%)	2 (16.7%)	0.906
Residential area, *n* (%)					0.786
Provincial capital	17 (26.2%)	52 (23.6%)	14 (16.9%)	3 (25.0%)	
Prefecture level city	6 (9.2%)	17 (7.7%)	5 (6.0%)	0 (0.0%)	
County level city	16 (24.6%)	61 (27.7%)	30 (36.1%)	5 (41.7%)	
County town	26 (40.0%)	90 (40.9%)	34 (41.0%)	4 (33.3%)	
Educational level, *n* (%)					0.624
Primary school or below	28 (43.1%)	112 (50.9%)	41 (49.4%)	5 (41.7%)	
Junior high school	19 (29.2%)	52 (23.6%)	17 (20.5%)	5 (41.7%)	
Senior high school	12 (18.5%)	30 (13.6%)	18 (21.7%)	1 (8.3%)	
College or above	6 (9.2%)	26 (11.8%)	7 (8.4%)	1 (8.3%)	
Serum albumin (g/L), mean ± SD	35.3 ± 5.7	36.6 ± 4.5	36.9 ± 4.7	37.2 ± 3.9	0.275
Hemoglobin (g/L), mean ± SD	115.2 ± 17.6 [*n* = 65]	118.6 ± 20.7 [*n* = 220]	126.0 ± 24.4 [*n* = 82]	127.4 ± 31.7 [*n* = 12]	**0.024**
KPS, median (IQR)	80.0 (80.0, 90.0)	90.0 (80.0, 90.0)	90.0 (80.0, 90.0)	90.0 (90.0, 90.0)	**<0.001**
Prealbumin (mg/L), mean ± SD	175.3 ± 62.5	194.6 ± 69.7	206.1 ± 70.7	228.3 ± 62.3	**0.015**
C-reactive protein (mg/L), median (IQR)	3.5 (3.2, 6.7) [*n* = 31]	3.5 (3.2, 15.6) [*n* = 101]	5.4 (3.2, 12.0) [*n* = 40]	NA (*n* = 0)	0.548
Platelet count (×10^9^/L), mean ± SD	239.8 ± 130.0	251.4 ± 95.4	263.8 ± 79.9	299.3 ± 116.3	0.292
Neutrophil to lymphocyte ratio, median (IQR)	2.1 (1.4, 3.5)	2.3 (1.6, 3.7)	2.4 (1.6, 3.2)	2.2 (1.7, 3.2)	0.689
Surgical procedure, *n* (%)					**<0.001**
Radical surgery	37 (56.9%)	85 (38.6%)	26 (31.3%)	1 (8.3%)	
Palliative resection	10 (15.4%)	15 (6.8%)	4 (4.8%)	0 (0.0%)	
Non-surgical	18 (27.7%)	120 (54.5%)	53 (63.9%)	11 (91.7%)	
Chemotherapy, yes, *n* (%)	51 (78.5%)	146 (66.4%)	51 (61.4%)	3 (25.0%)	**0.003**
Immunotherapy, yes, *n* (%)	2 (3.1%)	2 (0.9%)	2 (2.4%)	0 (0.0%)	0.421
Nutritional support, *n* (%)					0.149
None	51 (78.5%)	177 (80.5%)	78 (94.0%)	12 (100.0%)	
PN only	7 (10.8%)	23 (10.5%)	2 (2.4%)	0 (0.0%)	
EN and/or ONS only	6 (9.2%)	18 (8.2%)	3 (3.6%)	0 (0.0%)	
Combined PN and EN and/or ONS	1 (1.5%)	2 (0.9%)	0 (0.0%)	0 (0.0%)	
NRS2002, *n* (%)					**<0.001**
<3	0 (0.0%)	79 (35.9%)	30 (36.1%)	6 (50.0%)	
≥3	65 (100.0%)	141 (64.1%)	53 (63.9%)	6 (50.0%)	
PG-SGA, *n* (%)					**<0.001**
0–3	3 (4.6%)	45 (20.5%)	20 (24.1%)	4 (33.3%)	
4–8	26 (40.0%)	97 (44.1%)	39 (47.0%)	7 (58.3%)	
≥9	36 (55.4%)	78 (35.5%)	24 (28.9%)	1 (8.3%)	
Weight change over 1 month (%), median (IQR)	−5.0 (−8.9, 0.0) [*n* = 63]	−3.4 (−7.0, 0.0) [*n* = 209]	−3.1 (−6.8, 0.0) [*n* = 78]	0.0 (−1.6, 0.0) [*n* = 12]	0.048
Weight change over 6 months (%), median (IQR)	−12.8 (−15.8, −8.1) [*n* = 10]	−10.0 (−14.0, −5.8) [*n* = 24]	−7.1 (−8.2, −4.3) [*n* = 9]	NA (*n* = 0)	0.170
Weight loss ≥5% over 6 months, *n* (%) (*n* = 43)					0.492
No	1 (10.0%)	6 (25.0%)	3 (33.3%)	NA (*n* = 0)	
Yes	9 (90.0%)	18 (75.0%)	6 (66.7%)	NA (*n* = 0)	
Available n	10	24	9	0	
Vital status, *n* (%)					**0.010**
Alive/censored	18 (27.7%)	109 (49.5%)	41 (49.4%)	7 (58.3%)	
Deceased	47 (72.3%)	111 (50.5%)	42 (50.6%)	5 (41.7%)	

Percentages were calculated using non-missing observations within each BMI group and were rounded independently to one decimal place; totals may therefore not sum exactly to 100.0%. The available sample size (n) is reported for variables with missing data.

Values are presented as mean ± SD, median (IQR), or n (%). p values were derived using Welch’s one-way ANOVA for continuous variables presented as mean ± SD, the Kruskal-Wallis test for continuous variables presented as median (IQR), and the Fisher–Freeman–Halton test with 100,000 Monte Carlo samples for categorical variables. Comparisons for CRP and 6-month weight change were restricted to the three BMI groups with available observations.

Weight change (%) was calculated as 100 × (current recalled weight − previous recalled weight) / previous recalled weight; negative values indicate weight loss. Previous weights recorded as 0 were considered missing. Significant weight loss was defined as a weight reduction of ≥5% within 6 months.

Bold *p* values indicate p values of statistical significance (*p* < 0.05).

BMI, body mass index; CI, confidence interval; CRP, C-reactive protein; EN, enteral nutrition; HR, hazard ratio; IQR, interquartile range; KPS, Karnofsky Performance Status; NLR, neutrophil-to-lymphocyte ratio; ONS, oral nutritional supplements; PG-SGA, Patient-Generated Subjective Global Assessment; PN, parenteral nutrition; SD, standard deviation.

Age, sex, clinical TNM stage, family history of gastric cancer, smoking, alcohol consumption, residential area, educational level, serum albumin, and immunotherapy did not differ significantly across BMI categories (all *p* > 0.05). In contrast, hemoglobin levels (*p* = 0.024), KPS (*p* < 0.001), prealbumin levels (*p* = 0.015), and 1-month weight change (*p* = 0.048) differed across BMI groups.

Treatment characteristics also varied across BMI categories. The proportion of patients undergoing radical surgery decreased from 56.9% in the underweight group to 8.3% in the obese group, whereas the proportion of patients who did not undergo surgery increased across BMI categories (*p* < 0.001). Chemotherapy use also differed significantly among the four groups (*p* = 0.003).

Baseline nutritional status differed across BMI categories. All underweight patients had an NRS 2002 score ≥3, compared with 64.1% of normal-weight patients, 63.9% of overweight patients, and 50.0% of obese patients (*p* < 0.001). Similarly, severe nutritional impairment, defined by a PG-SGA score ≥9, was most prevalent among underweight patients (55.4%) and decreased progressively across the normal-weight (35.5%), overweight (28.9%), and obese (8.3%) groups (*p* < 0.001).

During follow-up (median follow-up, 37.0 months by reverse Kaplan–Meier estimation; observed range, 0.2–67.8 months), 205 deaths occurred in the overall cohort. Among the 374 patients included in the primary multivariable analysis, 202 died. The proportions of patients who died were 72.3%, 50.5%, 50.6%, and 41.7% in the underweight, normal-weight, overweight, and obese groups, respectively (*p* = 0.010; Table 1).

### 3.2 Univariable associations with overall survival

In univariable Cox regression analysis, overweight patients had a lower mortality hazard than normal-weight patients (HR, 0.65; 95% CI, 0.45–0.93; *p* = 0.020). Neither underweight nor obesity was significantly associated with OS. Age was not associated with mortality when modeled per 10-year increase (HR, 0.99; 95% CI, 0.88–1.10; *p* = 0.814; Table 2).

**Table 2:** Univariate analysis of nutritional and clinical data of patients with gastric cancer.

Characteristic	N	Deaths	HR	95% CI	*p* value
Sex	380	205			
Male	268	140	*Reference*	—	—
Female	112	65	1.21	0.90 to 1.62	0.216
Age, per 10 years	380	205	0.99	0.88 to 1.10	0.814
BMI (kg/m^2^)	380	205			
18.5–23.9	220	111	*Reference*	—	—
<18.5	65	47	1.19	0.85 to 1.68	0.316
24.0–27.9	83	42	0.65	0.45 to 0.93	**0.020**
≥28.0	12	5	0.49	0.20 to 1.21	0.124
TNM stage	374	202			
I	29	4	*Reference*	—	—
II	60	22	3.05	1.05 to 8.85	**0.041**
III	156	81	5.12	1.87 to 13.99	**0.001**
IV	129	95	9.79	3.59 to 26.66	**<0.001**
Family history	380	205			
No	341	183	*Reference*	—	—
Yes	39	22	0.93	0.60 to 1.45	0.762
Smoking	380	205			
No	223	122	*Reference*	—	—
Yes	157	83	1.08	0.82 to 1.43	0.595
Alcohol consumption	380	205			
No	320	174	*Reference*	—	—
Yes	60	31	0.92	0.63 to 1.36	0.679
PG-SGA	380	205			
0–3	72	35	*Reference*	—	—
4–8	169	85	1.12	0.75 to 1.66	0.585
≥9	139	85	1.65	1.11 to 2.46	**0.013**
NRS2002	380	205			
<3	115	62	*Reference*	—	—
≥3	265	143	0.96	0.72 to 1.30	0.810
KPS, per point	380	205	0.95	0.93 to 0.97	**<0.001**
Albumin, per 1 g/L	380	205	0.96	0.93 to 0.99	**0.004**
Prealbumin, per 1 mg/L	380	205	0.9952	0.9932 to 0.9973	**<0.001**
Hemoglobin, per 1 g/L	379	205	0.996	0.990 to 1.002	0.218
C-reactive protein, per 1 mg/L	172	81	1.000	0.993 to 1.006	0.877
NLR, per unit	380	205	1.09	1.04 to 1.14	**<0.001**
Platelets, per 1 × 10^9^/L	380	205	1.001	0.999 to 1.002	0.296
1-month weight change, per percentage point	362	194	0.985	0.956 to 1.014	0.305
6-month weight change, per percentage point	43	30	0.953	0.911 to 0.997	**0.035**
Weight loss ≥5% over 6 months	43	30			
No	10	4	*Reference*	—	—
Yes	33	26	3.20	1.10 to 9.30	**0.032**
Surgery in operated patients	178	90			
Radical surgery	149	69	*Reference*	—	—
Palliative resection	29	21	1.80	1.10 to 2.95	**0.019**
Chemotherapy	380	205			
No	129	59	*Reference*	—	—
Yes	251	146	1.56	1.15 to 2.11	**0.004**
Nutritional support	380	205			
None	318	163	*Reference*	—	—
PN only	32	22	2.05	1.31 to 3.21	**0.002**
EN and/or ONS only	27	19	2.05	1.27 to 3.32	**0.003**
Combined	3	1	0.32	0.05 to 2.32	0.262
Histological differentiation	236	126			
G1	4	3	*Reference*	—	—
G2	71	23	0.36	0.11 to 1.21	**0.098**
G3	160	99	0.91	0.29 to 2.88	0.870
G4	1	1	0.81	0.08 to 7.81	0.852

N and deaths refer to the observations included in each univariable model. Continuous-variable HRs are expressed per specified increment; age was analyzed per 10-year increase. Missing values were excluded on a model-specific basis.

Weight change (%) was defined in the methdology. Previous weights recorded as 0 were considered missing. Significant weight loss was defined as a weight reduction of ≥5% within 6 months.

Bold *p* values indicate *p* values of statistical significance (*p* < 0.05).

BMI, body mass index; CI, confidence interval; CRP, C-reactive protein; EN, enteral nutrition; HR, hazard ratio; IQR, interquartile range; KPS, Karnofsky Performance Status; NLR, neutrophil-to-lymphocyte ratio; ONS, oral nutritional supplements; PG-SGA, patient-generated subjective global assessment; PN, parenteral nutrition; SD, standard deviation.

Clinical TNM stage was strongly associated with mortality. Compared with stage I disease, the mortality hazard increased progressively across stages II (HR, 3.05; 95% CI, 1.05–8.85; *p* = 0.041), III (HR, 5.12; 95% CI, 1.87–13.99; *p* = 0.001), and IV (HR, 9.79; 95% CI, 3.59–26.66; *p* < 0.001).

Among nutritional and functional indicators, severe nutritional impairment, defined by a PG-SGA score ≥ 9 was associated with increased mortality compared with a score of 0–3 (HR, 1.65; 95% CI, 1.11–2.46; *p* = 0.013). In contrast, NRS2002-defined nutritional risk was not associated with OS (≥ 3 vs. < 3; HR, 0.96; 95% CI, 0.72–1.30; *p* = 0.810). Higher Karnofsky Performance Status (KPS) was associated with a lower mortality hazard (HR per one-point increase, 0.95; 95% CI, 0.93–0.97; *p* < 0.001).

Higher serum albumin and prealbumin levels were also associated with lower mortality hazards in univariable analyses. Higher neutrophil-to-lymphocyte ratio (NLR) was associated with increased mortality (HR, 1.09; 95% CI, 1.04–1.14; *p* < 0.001). Hemoglobin, C-reactive protein, platelet count, and 1-month weight change were not significantly associated with OS.

Among the 43 patients with available 6-month weight-loss data, significant weight loss of at least 5% was associated with increased mortality (HR, 3.20; 95% CI, 1.10–9.30; *p* = 0.032).

Treatment-related variables were also associated with survival. Among surgically treated patients, palliative resection was associated with higher mortality than radical surgery (HR, 1.80; 95% CI, 1.10–2.95; *p* = 0.019). Receipt of chemotherapy and parenteral or enteral nutritional support was also associated with higher mortality in univariable analyses (Table 2).

### 3.3 Multivariable analysis of prognostic factors

The multivariable Cox model included 374 patients and 202 deaths. The model included BMI category, age, TNM stage, PG-SGA category, KPS, prealbumin, NLR, and baseline chemotherapy, with the baseline hazard stratified according to surgical procedure and nutritional support type.

After multivariable adjustment, BMI 24.0–27.9 kg/m^2^ was associated with a lower mortality hazard than BMI 18.5–23.9 kg/m^2^ (HR, 0.62; 95% CI, 0.41–0.93; *p* = 0.020). Neither underweight (HR, 0.89; 95% CI, 0.60–1.34; *p* = 0.592) nor obesity (HR, 0.51; 95% CI, 0.20–1.30; *p* = 0.160) was significantly associated with OS.

Compared with stage I disease, stage III (HR, 4.63; 95% CI, 1.64–13.06; *p* = 0.004) and stage IV disease (HR, 10.93; 95% CI, 3.74–31.95; *p* < 0.001) were associated with higher mortality. Stage II disease was not significantly associated with mortality (HR, 2.84; 95% CI, 0.95–8.50; *p* = 0.062). Age was not significantly associated with mortality when modeled as a spline with four degrees of freedom (overall *p* = 0.304).

Higher prealbumin levels remained associated with a lower mortality hazard (HR per 10-mg/L increase, 0.972; 95% CI, 0.947–0.996; *p* = 0.023). Receipt of chemotherapy at baseline was associated with lower adjusted mortality (HR, 0.653; 95% CI, 0.427–0.997; *p* = 0.048). PG-SGA category, KPS, and NLR were not independently associated with OS (Table 3 and Figure 1). The final model had a Harrell C-index of 0.703.

**Table 3:** Multivariable cox regression for overall survival.

Characteristic	*N*	Deaths	Adjusted HR (95% CI)	*P* value
BMI (kg/m^2^)	374	202		
18.5–23.9	216	109	*Reference*	—
<18.5	64	46	0.89 (0.60 to 1.34)	0.592
24.0–27.9	82	42	0.62 (0.41 to 0.93)	0.020
≥28.0	12	5	0.51 (0.20 to 1.30)	0.160
Age, RCS (4 df)	374	202	—	0.304
TNM stage	374	202		
I	29	4	*Reference*	—
II	60	22	2.84 (0.95 to 8.50)	0.062
III	156	81	4.63 (1.64 to 13.06)	**0.004**
IV	129	95	10.93 (3.74 to 31.95)	**<0.001**
PG-SGA	374	202		
0–3	71	35	*Reference*	—
4–8	167	84	1.04 (0.68 to 1.60)	0.860
≥9	136	83	1.25 (0.79 to 1.96)	0.337
KPS, per 10 points	374	202	0.83 (0.64 to 1.09)	0.180
Prealbumin, per 10 mg/L	374	202	0.972 (0.947 to 0.996)	**0.023**
NLR, per unit	374	202	1.01 (0.95 to 1.07)	0.801
Chemotherapy	374	202		
No	127	59	*Reference*	—
Yes	247	143	0.653 (0.427 to 0.997)	**0.048**
Stratification factors				
Surgical procedure	374	202	Stratified	—
Nutritional support	374	202	Stratified	—

The model includes BMI category, age (restricted cubic spline, 4 df), TNM stage, PG-SGA category, KPS, prealbumin, NLR, and baseline chemotherapy, with baseline hazards stratified jointly by surgical procedure and nutritional support. All estimates use the same 374 complete cases and 202 deaths. The global proportional-hazards test was not significant (*p* = 0.346).

Bold *p* values indicate *p* values of statistical significance (*p* < 0.05).

BMI, body mass index; CI, confidence interval; CRP, C-reactive protein; EN, enteral nutrition; HR, hazard ratio; IQR, interquartile range; KPS, Karnofsky Performance Status; NLR, neutrophil-to-lymphocyte ratio; ONS, oral nutritional supplements; PG-SGA, Patient-Generated Subjective Global Assessment; PN, parenteral nutrition; SD, standard deviation.

**Figure 1:**
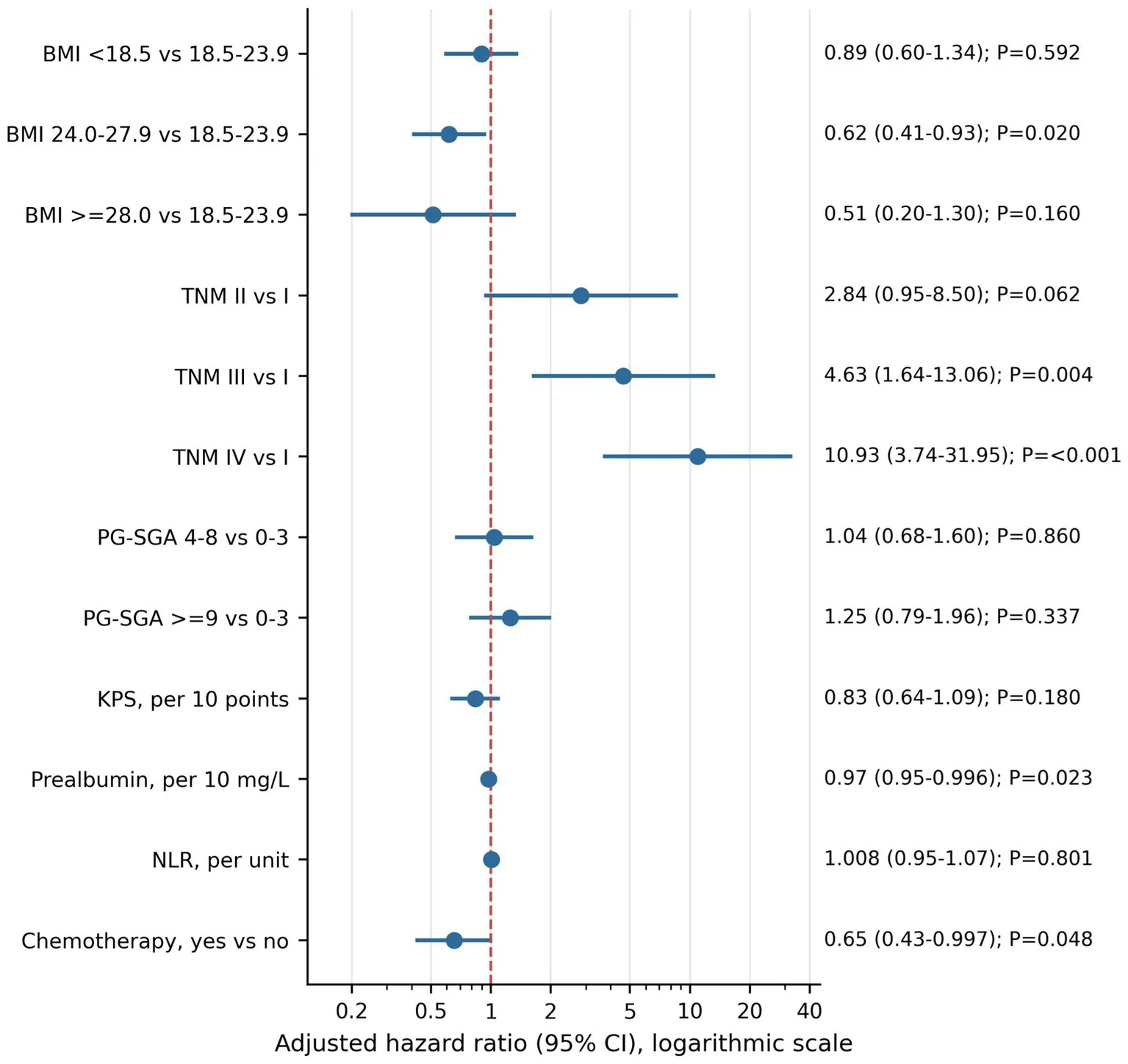
Multivariable Cox regression model for overall survival. Hazard ratios and 95% confidence intervals were derived from the final multivariable Cox model. Age was modeled using a restricted cubic spline and therefore is not represented by a single hazard ratio. The baseline hazard was stratified by surgical procedure and nutritional support type. Proportional hazards assumptions were assessed using score tests based on the Kaplan–Meier transformation of time. No evidence of violation was observed for any BMI category (all *p* ≥ 0.172) or for the model overall (χ^2^ = 17.62, 16 df; *p* = 0.346).

### 3.4 Stage-stratified association between BMI and overall survival

In the parsimonious early-stage model (89 patients; 26 deaths), underweight was associated with higher mortality than normal weight (HR, 5.19; 95% CI, 1.42–18.96; *p* = 0.013). Overweight (HR, 1.47; 95% CI, 0.53–4.08; *p* = 0.462) and obesity (HR, 0.74; 95% CI, 0.08–6.69; *p* = 0.791) were not significantly associated with mortality.

In the parsimonious advanced-stage model (285 patients; 176 deaths), overweight was associated with lower mortality (HR, 0.61; 95% CI, 0.41–0.93; *p* = 0.021). Underweight (HR, 0.86; 95% CI, 0.58–1.26; *p* = 0.426) and obesity (HR, 0.39; 95% CI, 0.14–1.06; *p* = 0.064) were not significantly associated with mortality.

Within each stage stratum, more advanced TNM stage remained associated with higher mortality. Stage II versus stage I was associated with an HR of 3.19 (95% CI, 1.04–9.76; *p* = 0.042), whereas stage IV versus stage III was associated with an HR of 1.86 (95% CI, 1.37–2.52; *p* < 0.001). The BMI-by-stage interaction was not statistically significant (*p* = 0.173; Table 4 and Figure 2).

**Table 4:** Parsimonious stage-specific sensitivity analyses for overall survival.

Group	Characteristic	Adjusted HR (95% CI)	*P* value
Early stage I-II *N* = 89; deaths = 26	BMI category		
	18.5–23.9	*Reference*	—
	<18.5	5.19 (1.42 to 18.96)	**0.013**
	24.0–27.9	1.47 (0.53 to 4.08)	0.462
	≥28.0	0.74 (0.08 to 6.69)	0.791
	TNM stage		
	I	*Reference*	—
	II	3.19 (1.04 to 9.76)	**0.042**
Advanced stage III-IV *N* = 285; deaths = 176	BMI category		
	18.5–23.9	*Reference*	—
	<18.5	0.86 (0.58 to 1.26)	0.426
	24.0–27.9	0.61 (0.41 to 0.93)	**0.021**
	≥28.0	0.39 (0.14 to 1.06)	**0.064**
	TNM stage		
	III	*Reference*	—
	IV	1.86 (1.37 to 2.52)	**<0.001**

Parsimonious sensitivity models included BMI category and within-stratum TNM stage, with baseline hazards stratified by age group (<60, 60–69, or ≥70 years). The early-stage and advanced-stage analyses included 89 patients/26 deaths and 285 patients/176 deaths, respectively. The formal categorical BMI-by-stage interaction test in the full final model was not significant (*p* = 0.173). Bold *p* values indicate *p* values of statistical significance (*p* < 0.05).

**Figure 2:**
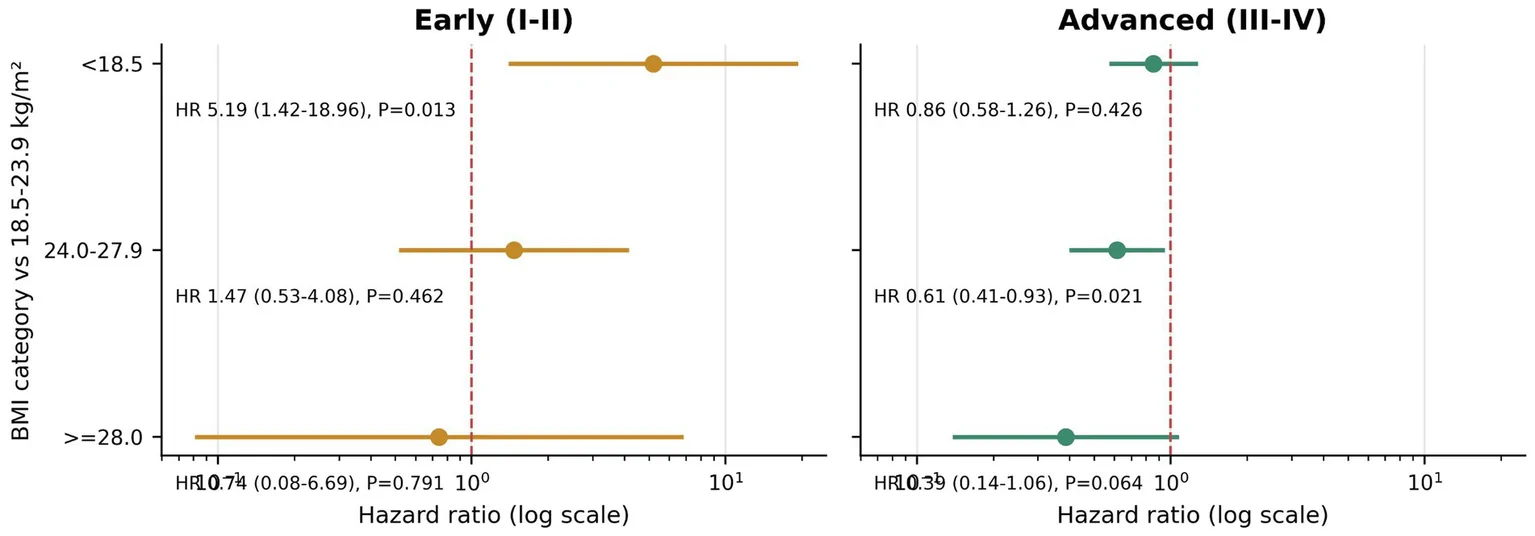
BMI estimates from parsimonious stage-specific sensitivity analyses. The forest plot presents age-stratified parsimonious sensitivity estimates for BMI categories within early-stage (I–II) and advanced-stage (III–IV) disease. These subgroup estimates are exploratory; the formal BMI-by-stage interaction was not significant (*p* = 0.173).

In the parsimonious early-stage sensitivity model (89 patients; 26 deaths), underweight was associated with higher mortality than normal weight (HR, 5.19; 95% CI, 1.42–18.96; *p* = 0.013). Estimates for overweight (HR, 1.47; 95% CI, 0.53–4.08; *p* = 0.462) and obesity (HR, 0.74; 95% CI, 0.08–6.69; *p* = 0.791) were imprecise.

### 3.5 Dose–response associations of BMI and prealbumin with mortality

Restricted cubic spline analysis showed no significant overall association between continuous BMI and mortality (P_overall_ = 0.640) and no evidence of a nonlinear association (P_nonlinearity_ = 0.739; Figure 3). The adjusted hazard ratio was estimated relative to a BMI of 22 kg/m^2^.

**Figure 3:**
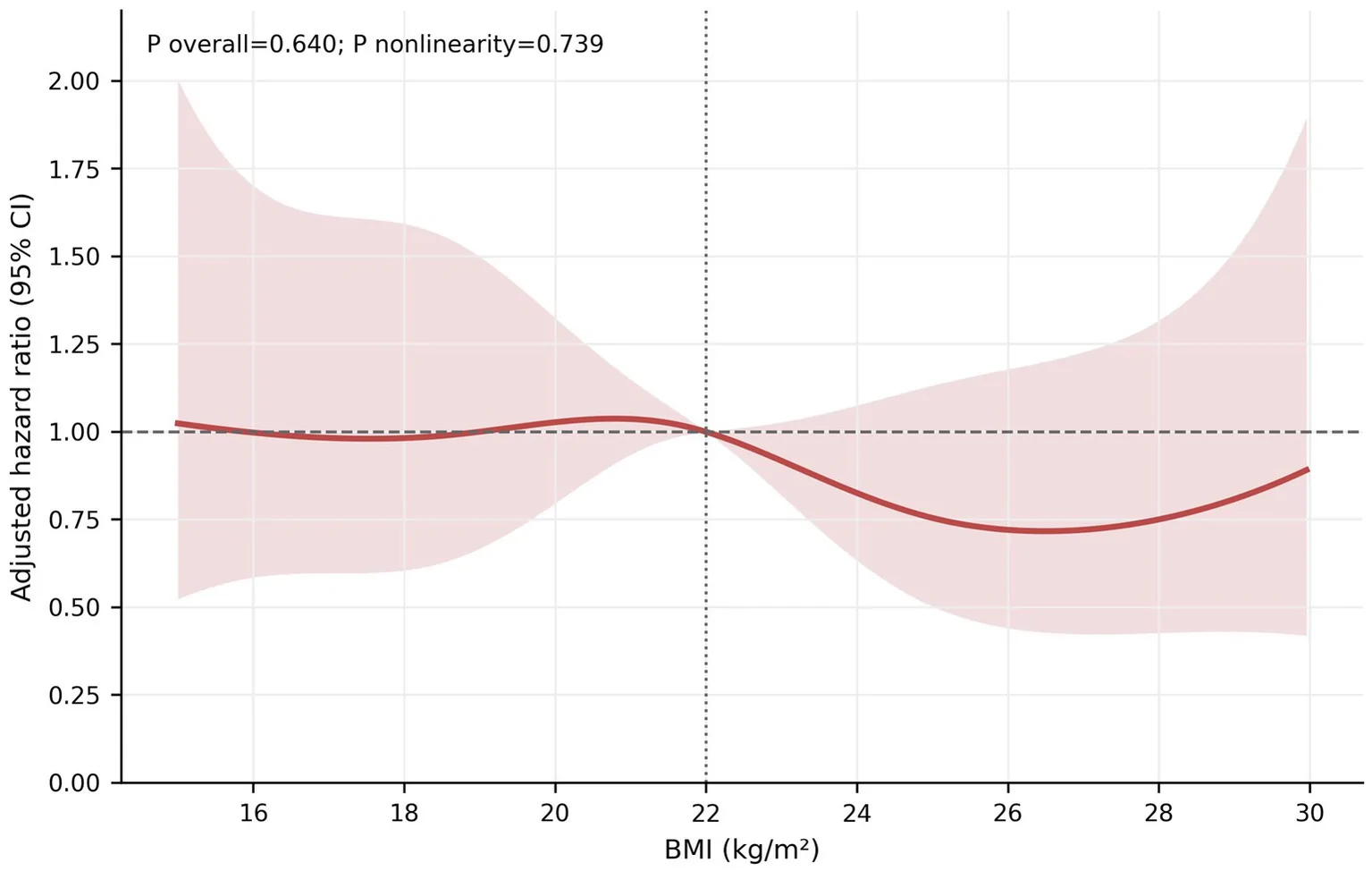
Restricted cubic spline analysis of continuous BMI and mortality. The solid line and shaded band show the adjusted hazard ratio and 95% confidence interval, with BMI 22 kg/m^2^ as the reference. Neither the overall association nor the nonlinear component was statistically significant.

For serum prealbumin, restricted cubic spline analysis showed no significant overall association with mortality (P_overall_ = 0.072) and no evidence of a nonlinear association (P_nonlinearity_ = 0.342; Figure 4). The adjusted hazard ratio was estimated relative to a prealbumin concentration of 194.5 mg/L.

**Figure 4:**
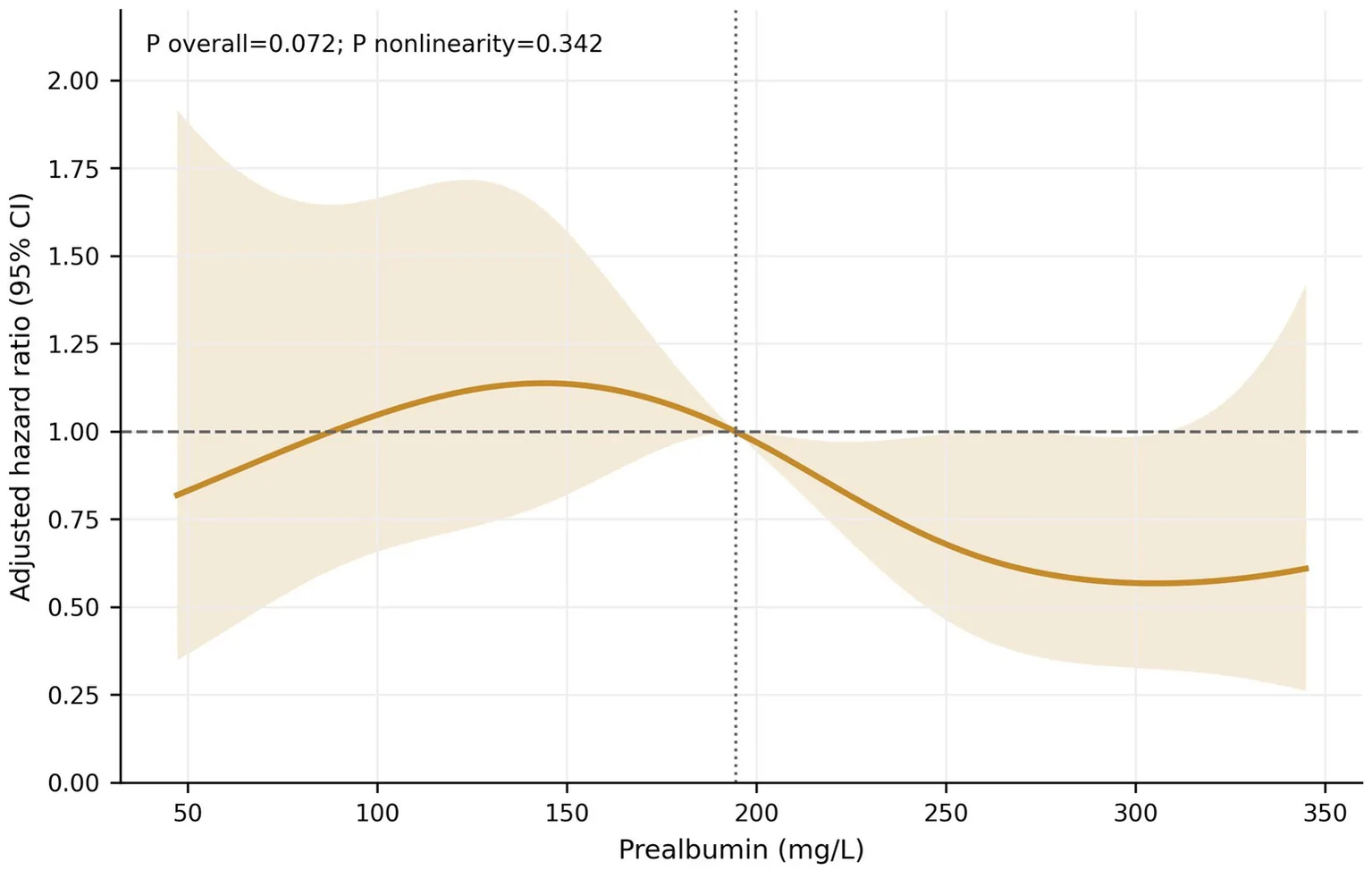
Restricted cubic spline analysis of continuous prealbumin and mortality. The solid line and shaded band show the adjusted hazard ratio and 95% confidence interval, with prealbumin 194.5 mg/L as the reference. Neither the overall association nor the nonlinear component was statistically significant. The spline findings should be interpreted together with the categorical and linear estimates because estimates at extreme BMI and prealbumin values were imprecise.

### 3.6 Kaplan–Meier survival analyses according to nutritional indicators

Kaplan–Meier analysis showed a significant overall difference in OS across the four BMI categories (log-rank *p* = 0.011; Figure 5). Unadjusted survival was lowest in the underweight group and higher across the normal-weight, overweight, and obese groups.

**Figure 5:**
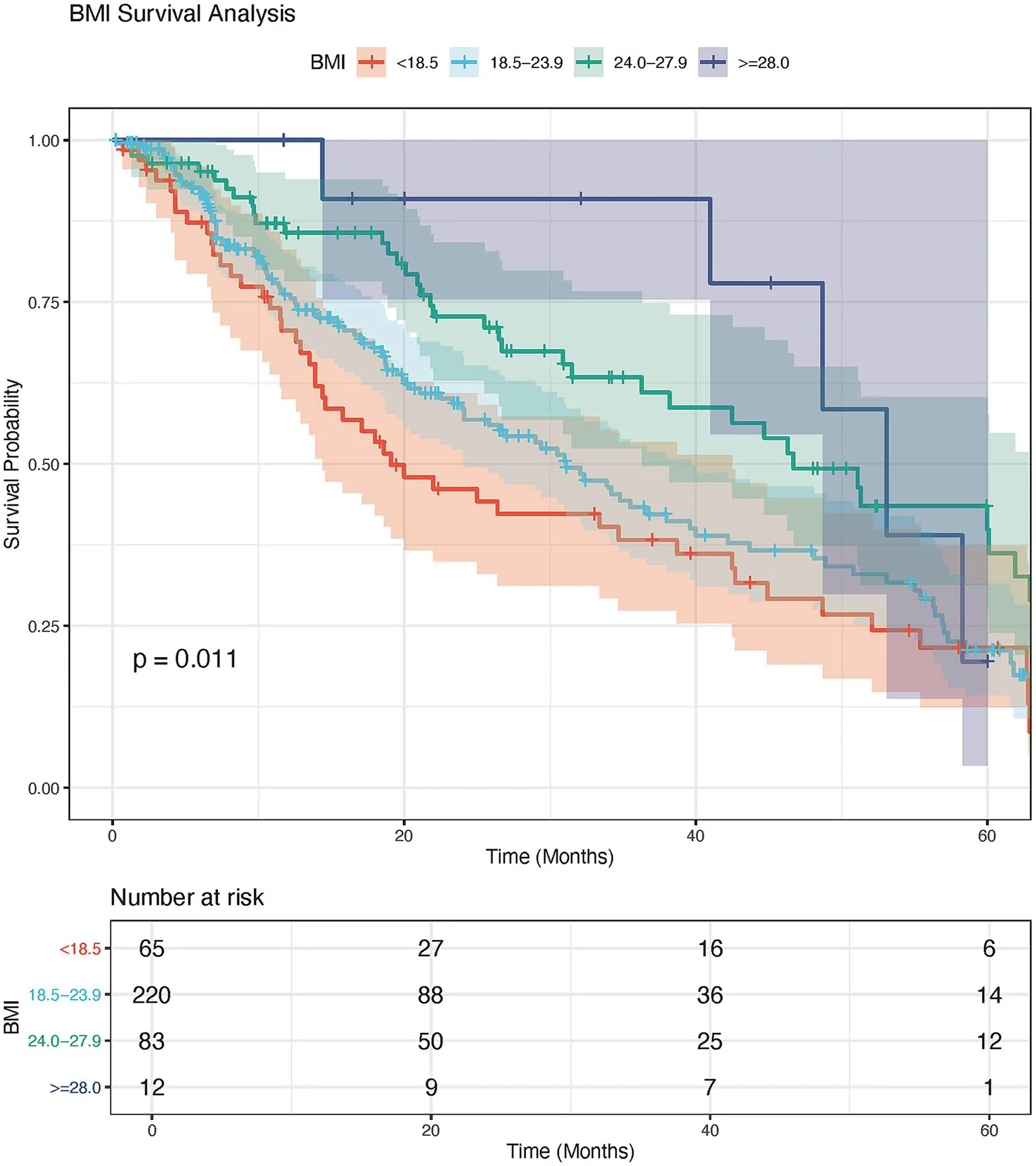
Kaplan–Meier survival curves for overall survival (OS) stratified by Body Mass Index (BMI) categories. The survival probability of patients with gastric cancer over a 60-month follow-up period is depicted for four BMI groups: <18.5 (underweight), 18.5–23.9 (normal), 24.0–27.9 (overweight), and > = 28.0 (obese). Shaded regions indicate the 95% confidence intervals for each survival curve. A log-rank test confirms a statistically significant difference in survival outcomes among the BMI cohorts (*p* = 0.011). The “Number at risk” table at the bottom of the figure displays the number of patients remaining in the study at 20-month intervals for each category.

In contrast, nutritional risk defined by NRS 2002 was not associated with a significant difference in OS. Survival curves were similar between patients with NRS 2002 scores <3 and ≥3 (log-rank *p* = 0.80; Figure 6).

**Figure 6:**
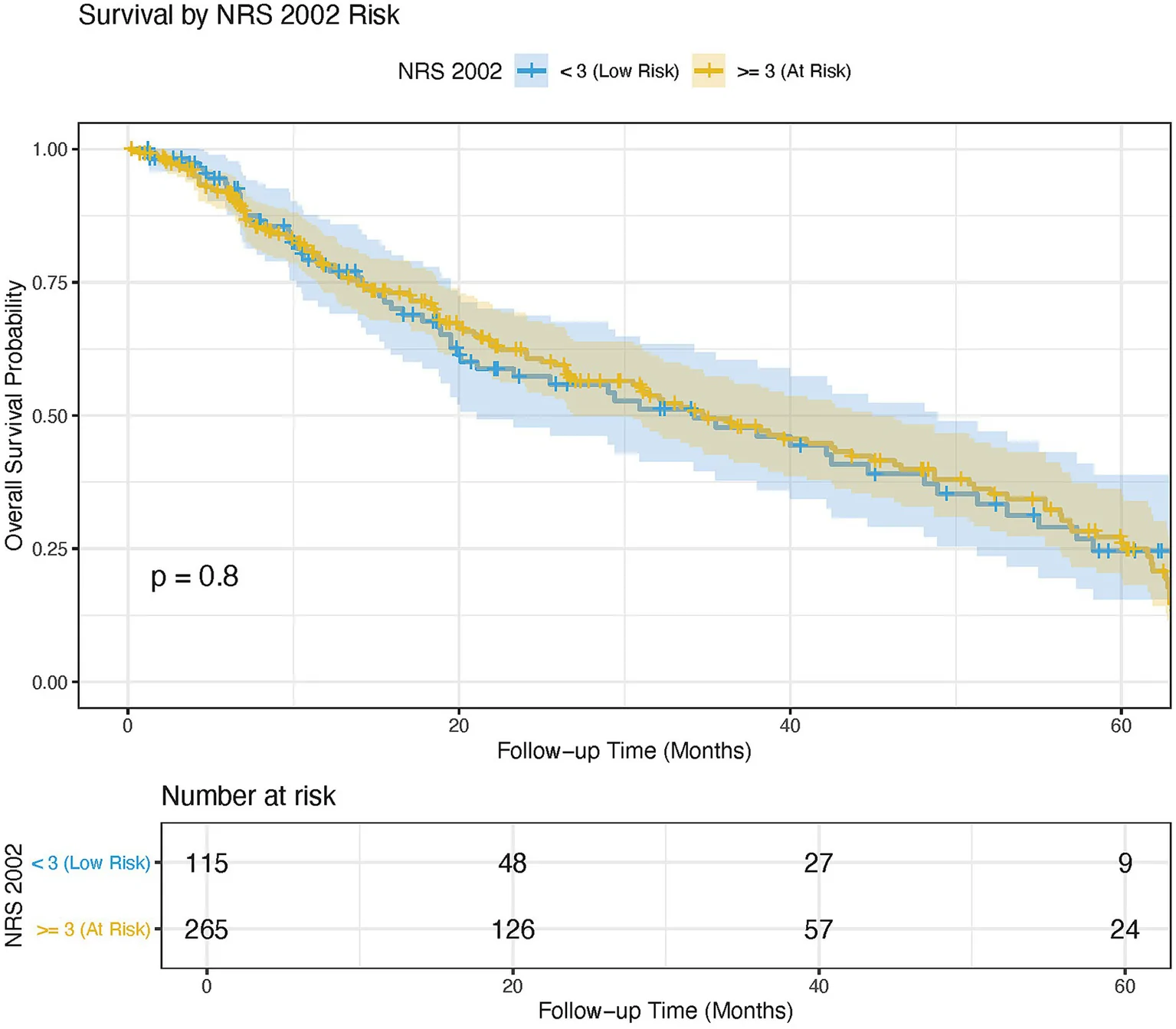
Kaplan–Meier survival curves for overall survival (OS) stratified by Nutritional Risk Screening 2002 (NRS2002) risk categories. The survival probability is compared between the Low Risk (score < 3, blue line) and At Risk (score > = 3, yellow line) groups over a 60-month follow-up period. Shaded regions represent the corresponding 95% confidence intervals. No statistically significant difference in survival outcomes was observed between the two risk groups (log-rank *p* = 0.8). The “Number at risk” table at the bottom indicates the remaining patient count at 20-month intervals for each cohort.

OS also differed significantly across the three PG-SGA categories in unadjusted Kaplan–Meier analysis (log-rank *p* = 0.0097; Figure 7). Higher PG-SGA scores were associated with poorer unadjusted survival. However, PG-SGA was not independently associated with OS in the multivariable Cox model.

**Figure 7:**
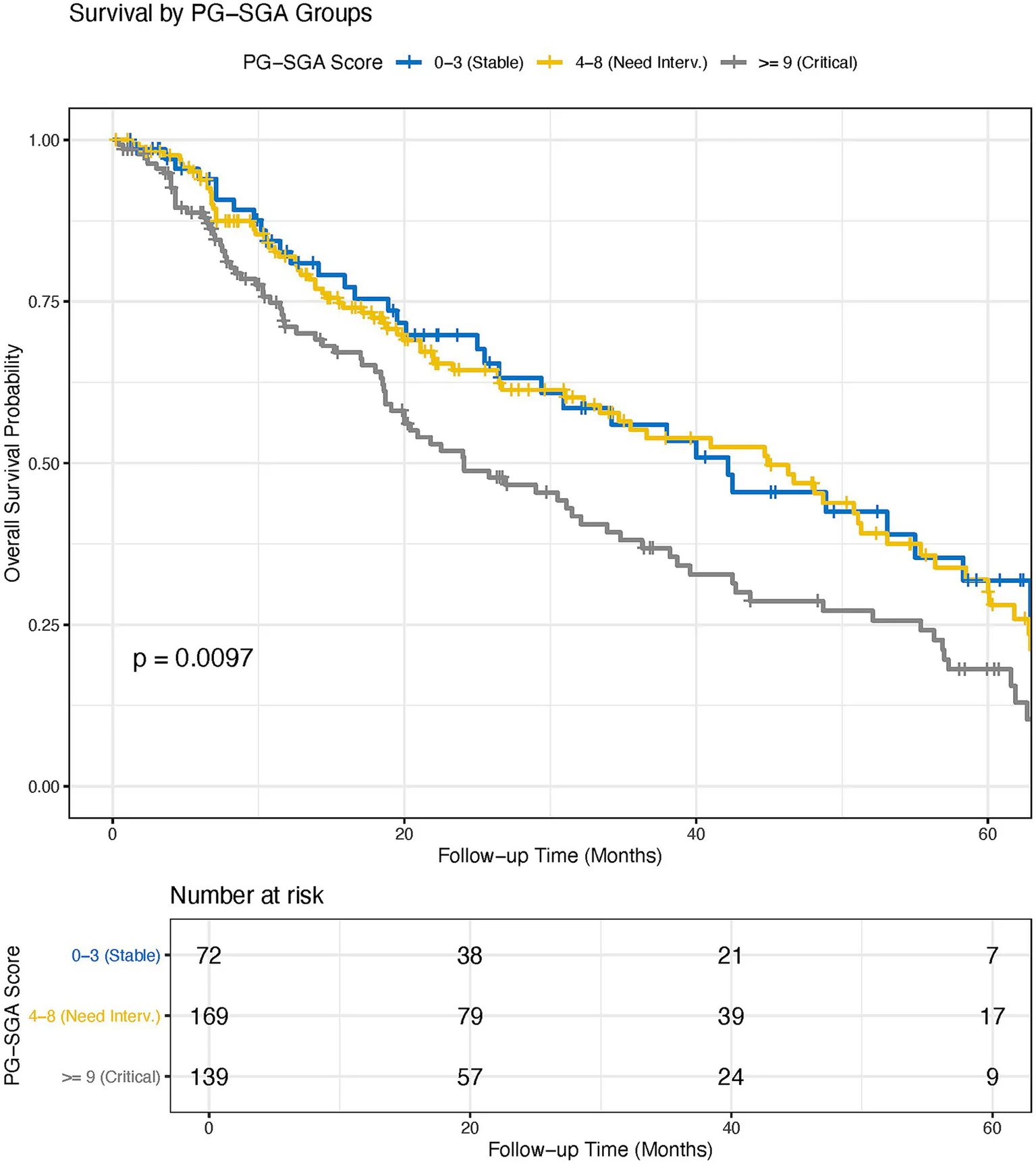
Kaplan–Meier survival curves for overall survival (OS) stratified by Patient-Generated Subjective Global Assessment (PG-SGA) categories. Patients were grouped according to PG-SGA scores of 0–3 (relatively stable nutritional status; *n* = 72), 4–8 (nutritional intervention indicated; *n* = 169), and ≥9 (urgent nutritional intervention indicated; *n* = 139). Shaded regions indicate the 95% confidence intervals. Overall survival differed significantly among the three groups (log-rank *p* = 0.0097). The number-at-risk table shows the patients remaining at risk at 0, 20, 40, and 60 months.

### 3.7 Development of an exploratory nutritional prognostic model

The clinical reference model included age, sex, TNM stage, and baseline chemotherapy. The extended clinical-nutritional model additionally included continuous BMI, PG-SGA score, KPS, serum prealbumin, and NLR, and was presented as a nomogram (Figure 8). The extended model showed improved model fit compared with the clinical reference model (likelihood-ratio χ^2^ = 21.80, 5 df; *p* < 0.001).

**Figure 8:**
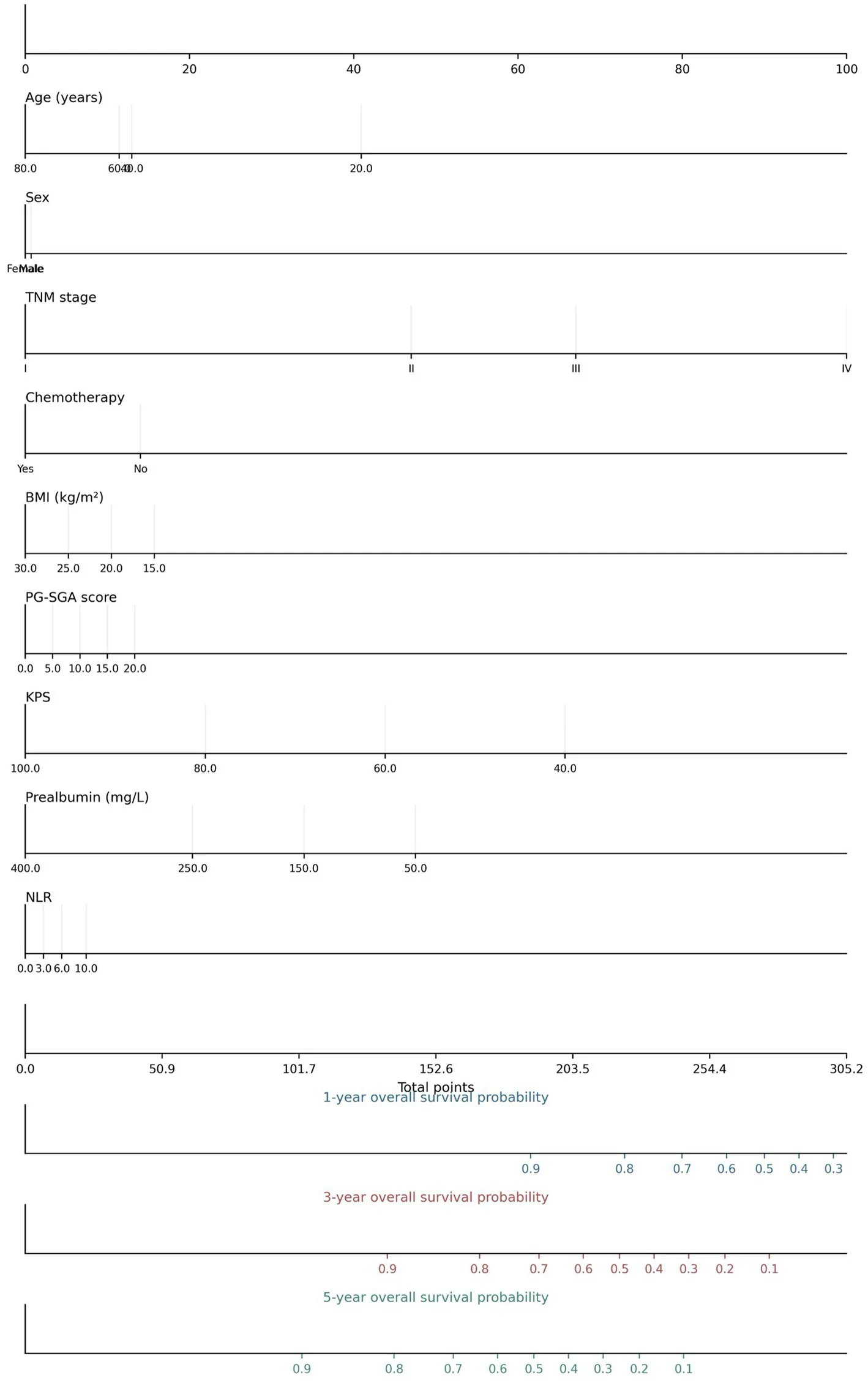
Extended clinical-nutritional nomogram for 1-, 3-, and 5-year OS. The nomogram displays the variables in the extended model: age, sex, TNM stage, baseline chemotherapy, BMI, PG-SGA score, KPS, prealbumin, and NLR. Point totals map to estimated 1-, 3-, and 5-year OS probabilities. It is intended for methodological illustration and not for clinical use without external validation. OS, overall survival; PG-SGA, Patient-Generated Subjective Global Assessment; NLR, Neutrophil-to-Lymphocyte Ratio; KPS, Karnofsky Performance Status.

The apparent C-index was 0.664 for the clinical model and 0.705 for the extended model. After 1,000 bootstrap resamples, the optimism-corrected C-indices were 0.646 and 0.681, respectively. Time-dependent AUCs for the clinical versus extended models were 0.689 versus 0.744 at 1 year, 0.715 versus 0.748 at 3 years, and 0.684 versus 0.702 at 5 years (Figure 9).

**Figure 9:**
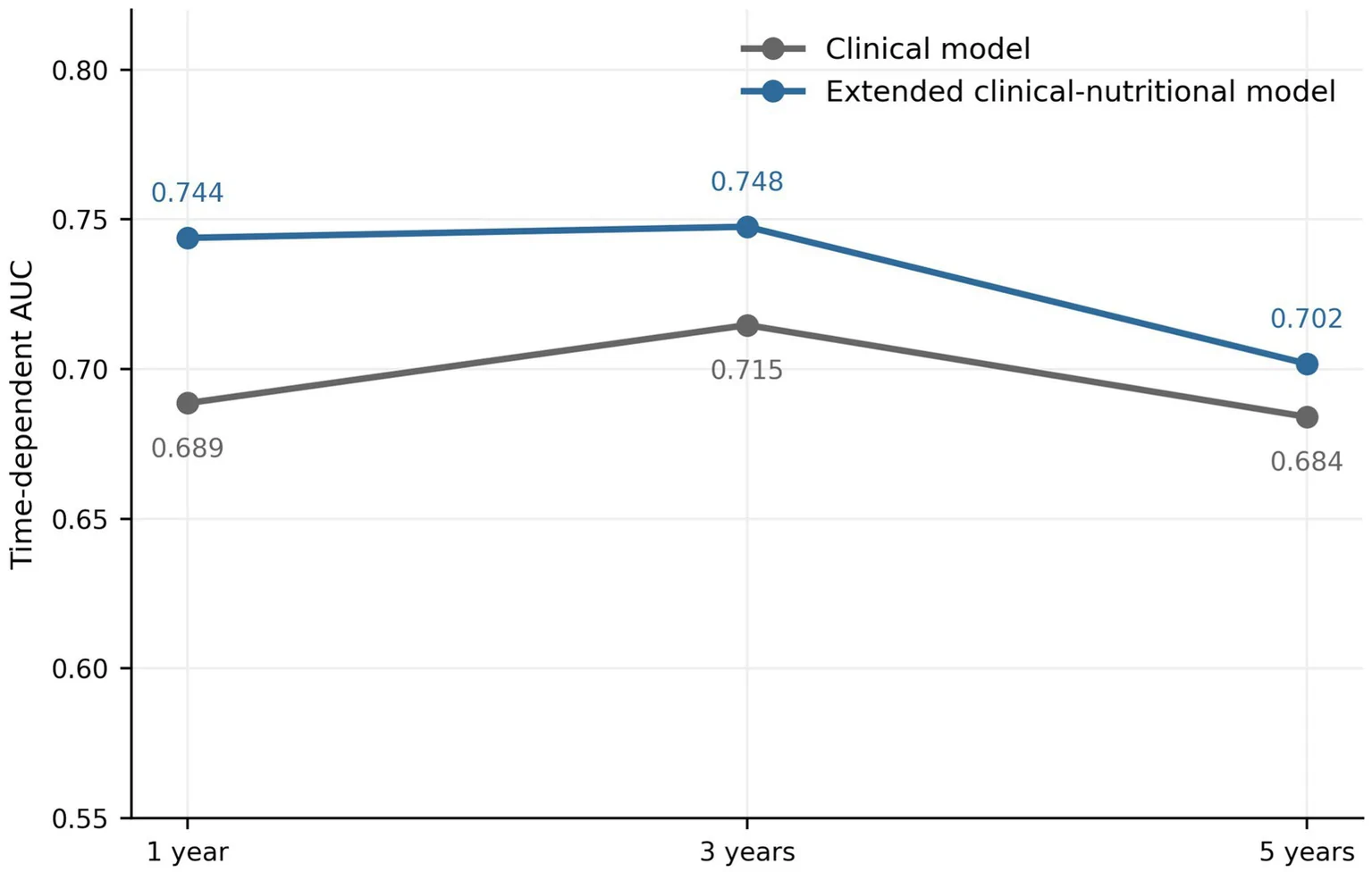
Time-dependent AUC comparison of the clinical and extended models. Time-dependent discrimination of the clinical reference and extended clinical-nutritional models at 1, 3, and 5 years. Values beside the points are the corresponding AUC estimates. AUC, area under the curve; OS, overall survival; ROC, receiver operating characteristic.

Calibration of predicted versus observed survival is shown for both models at 1, 3, and 5 years (Figure 10). Calibration was generally acceptable at 1 and 3 years but showed greater variability at 5 years.

**Figure 10:**
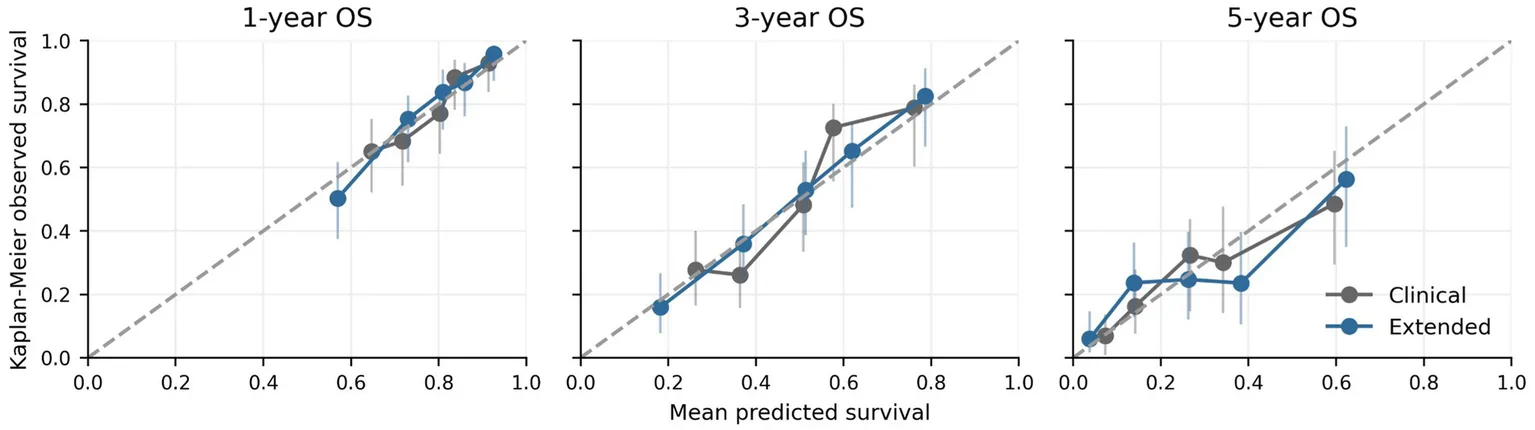
Calibration of the clinical and extended models. Mean predicted survival is plotted against Kaplan–Meier-observed survival within quintiles of predicted risk for the clinical and extended models. Error bars are 95% confidence intervals; the dashed diagonal denotes ideal calibration. AUC, area under the curve; BMI, body mass index; KPS, Karnofsky Performance Status; NLR, neutrophil-to-lymphocyte ratio; OS, overall survival; PG-SGA, Patient-Generated Subjective Global Assessment.

## 4 Discussion

In this observational cohort, BMI 24.0–27.9 kg/m^2^ was associated with a lower adjusted mortality hazard than BMI 18.5–23.9 kg/m^2^. The association was also observed in the advanced-stage subgroup, although the formal BMI-by-stage interaction was not statistically significant. PG-SGA distinguished unadjusted survival, whereas NRS 2002 did not. An extended clinical-nutritional prognostic model incorporating BMI and additional nutritional indicators showed better discrimination than the clinical reference model after internal validation.

The observed survival advantage associated with BMI in the overweight range is broadly consistent with recent evidence suggesting that the relationship between BMI and cancer prognosis may differ from the conventional association between excess body weight and cancer incidence. A 2024 systematic review and meta-analysis of cohort studies reported that underweight and weight loss were generally associated with poorer survival in patients with cancer, particularly among those with gastrointestinal malignancies, although substantial heterogeneity existed across tumor types and clinical settings (6). In gastric cancer specifically, recent observational studies have also described more favorable survival among patients with relatively high BMI (12). A study of patients with stage II–III gastric cancer undergoing radical gastrectomy found that BMI was associated with OS and that its prognostic implications could be influenced by perioperative chemotherapy (7). Similarly, a large postoperative cohort reported an apparent obesity paradox after curative gastrectomy, although the magnitude of the association varied across clinical subgroups (8). Our findings extend this literature to a clinically heterogeneous cohort encompassing different TNM stages and treatment pathways.

Several mechanisms may contribute to the observed association between higher BMI and improved survival. Gastric cancer commonly compromises food intake through anorexia, early satiety, obstruction, nausea, altered digestion, and treatment-related toxicity (5). In addition, systemic inflammation and tumor-driven metabolic changes may accelerate the loss of both skeletal muscle and adipose tissue. Patients with greater baseline energy and tissue reserves may therefore be better able to withstand progressive nutritional stress and treatment-related catabolism (13). Higher BMI may also be associated with a lower probability of having already entered an advanced cachectic state at the time of assessment. The adverse prognostic effects of cancer-associated weight loss and cachexia have been consistently documented, although definitions and body-composition measures vary across studies (2, 3).

Nutritional deterioration in gastric cancer may also result from potentially reversible barriers to oral intake. Malignant gastric outlet obstruction can cause vomiting, early satiety, and impaired oral feeding, while available treatments—including endoscopic stenting, endoscopic ultrasound-guided gastroenterostomy, and surgical gastrojejunostomy—differ in durability, reintervention requirements, and adverse-event profiles (14). In patients with combined biliary and gastric outlet obstruction, therapeutic endoscopic ultrasound may facilitate coordinated decompression and restoration of enteral intake (15).

Postoperative complications may further impair nutrition. Anastomotic strictures after esophagogastric surgery can reduce food passage and contribute to weight loss, while delayed gastric conduit emptying after esophagectomy may prolong intolerance of oral feeding and require endoscopic intervention (16, 17). Although the latter evidence is derived primarily from post-esophagectomy cohorts, these studies illustrate how structural and treatment-related gastrointestinal dysfunction can influence nutritional status. Together, these findings support interpreting baseline BMI as a marker shaped not only by body size and physiological reserve, but also by disease burden and the ability to maintain adequate oral intake.

BMI should therefore be interpreted as a readily accessible proxy for body size and physiological reserve rather than as a direct biological determinant of survival. It cannot distinguish skeletal muscle from adipose tissue, identify visceral versus subcutaneous adiposity, or detect sarcopenic obesity. Two patients with the same BMI may consequently have markedly different body composition and metabolic profiles (18). This limitation is particularly relevant in gastric cancer, in which low skeletal muscle mass may occur even among patients with normal or elevated BMI (19). Recent evidence indicates that sarcopenia and sarcopenic obesity provide prognostic information not fully captured by BMI, and computed tomography-derived body-composition measurements may improve risk stratification (20). Among patients with gastric cancer and cachexia, CT-defined low skeletal muscle mass has also been associated with poorer survival (21).

Although these findings are compatible with the frequently described “obesity paradox,” the association in our cohort was confined to the overweight range. The BMI ≥ 28.0 kg/m^2^ group was small, and its confidence interval included the null. The present results therefore primarily support an association between overweight BMI and survival rather than a broader survival advantage associated with obesity. Reverse causation may also contribute to this pattern. Patients with aggressive disease may experience substantial prediagnostic weight loss and consequently move from a previously higher BMI category into the normal-weight or underweight range. A study of upper gastrointestinal cancers showed that weight loss may provide more prognostic information than BMI measured at diagnosis and highlighted the importance of prediagnostic weight trajectories when interpreting inverse associations between BMI and mortality (10). Residual confounding by tumor burden, systemic inflammation, comorbidity, performance status, and treatment selection may also influence this relationship.

Stage-stratified analyses provided additional context. In patients with stage III–IV disease, overweight was associated with lower mortality, a clinical setting characterized by greater inflammatory burden, reduced intake, and progressive tissue wasting (22), whereas underweight was associated with higher mortality in the early-stage subgroup. However, the BMI-by-stage interaction was not statistically significant, indicating insufficient evidence that the association between BMI and mortality differed by TNM stage. The early-stage subgroup included only 26 deaths, resulting in a wide confidence interval for the underweight estimate. Larger studies are needed to determine whether the prognostic implications of BMI differ across disease stages.

An additional finding was the absence of an association between NRS 2002-defined nutritional risk and OS. Approximately 70% of the cohort had an NRS 2002 score ≥3, potentially limiting prognostic discrimination by placing a large proportion of patients within a single risk category. NRS 2002 was developed primarily to identify patients who may benefit from nutritional intervention rather than as a tumor-specific long-term survival model. Its performance as a nutritional screening instrument may therefore differ from its ability to discriminate long-term mortality risk.

By comparison, PG-SGA categories distinguished OS in Kaplan–Meier analysis. PG-SGA incorporates several dimensions relevant to cancer prognosis, including weight change, dietary intake, nutrition-impact symptoms, and functional impairment (23). It may therefore capture a broader spectrum of disease-related nutritional burden than BMI alone or a dichotomized screening score. Recent comparative evidence in oncology has suggested that PG-SGA identifies nutritional impairment more comprehensively than NRS 2002, although the tools were designed for partly different purposes (11). In the present study, however, the association between PG-SGA and mortality was attenuated after multivariable adjustment, suggesting substantial overlap with tumor stage, functional status, biochemical measures, and other indicators of disease severity.

The laboratory and functional findings further illustrate the overlap among nutritional status, inflammation, and disease severity. Higher albumin and prealbumin levels and better KPS were associated with lower mortality in univariable analyses, whereas higher NLR was associated with increased mortality. After multivariable adjustment, most of these associations were attenuated. Serum albumin and prealbumin are influenced not only by nutrient intake but also by systemic inflammation, hepatic synthesis, fluid status, and tumor burden. NLR similarly reflects systemic inflammation and may correlate with advanced stage and metastatic burden. KPS captures functional impairment that may also overlap with components assessed by PG-SGA and with factors influencing treatment selection. These correlations may partly account for the attenuation of individual nutritional and inflammatory markers in multivariable analysis.

Treatment-related associations also changed after adjustment. Chemotherapy was associated with higher mortality in univariable analysis but with lower mortality in the multivariable model. This reversal is consistent with substantial differences in clinical characteristics and treatment selection between patients who did and did not receive chemotherapy. The chemotherapy variable represented treatment already initiated by the baseline assessment and did not specifically denote postoperative adjuvant therapy. Associations involving nutritional support should be interpreted similarly, because the need for enteral or parenteral support is closely related to disease severity and impaired oral intake (24).

The exploratory prediction analysis compared an extended clinical-nutritional model with a clinical reference model containing age, sex, TNM stage, and baseline chemotherapy. Addition of BMI, PG-SGA, KPS, prealbumin, and NLR improved model fit and increased the optimism-corrected C-index from 0.646 to 0.681. The gains in time-dependent AUC were larger at 1 year than at 5 years, and calibration was less stable at the longest horizon. Together, these findings suggest that nutritional and functional variables provide incremental prognostic information beyond conventional clinical characteristics, although the magnitude of improvement was modest.

From a practical perspective, BMI remains attractive because it is inexpensive, objective, and routinely available. Its prognostic value may be greatest when interpreted as one component of a broader nutritional assessment rather than as an isolated marker. Recent weight loss, PG-SGA, functional status, inflammatory indicators, and, when available, CT-based skeletal muscle measurements may provide complementary information (25, 26). A multidimensional approach may better identify patients with reduced physiological reserve or ongoing tissue loss who could benefit from further nutritional assessment and supportive management.

Strengths of this study include the simultaneous evaluation of anthropometric, nutritional, inflammatory, functional, and treatment-related variables; formal assessment of the BMI-by-stage interaction; stage-stratified sensitivity analyses; and internal bootstrap validation of the prognostic models. The use of categorical, continuous, Kaplan–Meier, Cox regression, and spline analyses also allowed the BMI-survival association to be examined from complementary perspectives.

Several limitations should be acknowledged. First, the observational design precludes causal inference, and residual confounding cannot be excluded. Second, recruitment spanned a long period during which staging and treatment practices may have changed. Third, BMI was measured at one time point and cannot distinguish skeletal muscle from adipose tissue or characterize longitudinal changes in nutritional status. Fourth, the obesity group was small, limiting the precision of estimates for this category. Fifth, the early-stage subgroup included only 26 deaths. Sixth, CRP, tumor differentiation, and 6-month weight loss were available only for subsets of patients. Finally, the prognostic model was internally validated but requires evaluation in an independent cohort before its generalizability can be established.

In conclusion, BMI 24.0–27.9 kg/m^2^ was associated with a lower adjusted mortality hazard in patients with gastric cancer. Although a similar association was observed in patients with stage III–IV disease, there was no statistically significant BMI-by-stage interaction. PG-SGA, NRS 2002, biochemical markers, and functional measures provided different and partly overlapping information regarding nutritional status and prognosis. The addition of nutritional and functional variables modestly improved prognostic discrimination beyond conventional clinical factors. Future prospective studies incorporating longitudinal weight change, detailed body-composition measurements, and external validation may clarify the role of nutritional phenotyping in prognostic assessment for gastric cancer.

## Data Availability

The original contributions presented in the study are included in the article/supplementary material, further inquiries can be directed to the corresponding author/s.
